# Translation of metal-phthalocyanines adsorbed on Au(111): from van der Waals interaction to strong electronic correlation

**DOI:** 10.1038/s41598-018-31147-5

**Published:** 2018-08-24

**Authors:** L. Buimaga-Iarinca, C. Morari

**Affiliations:** National Institute for Research and Development of Isotopic and Molecular Technologies, 67-103 Donat, 400293 Cluj-Napoca, Romania

## Abstract

Using first-principles calculations, we investigate the binding energy for six transition metal - phthalocyanine molecules adsorbed on Au(111). We focus on the effect of translation on molecule - surface physical properties; van der Waals interactions as well as the strong correlation in *d* orbitals of transition metals are taken into account in all calculations. We found that dispersion interaction and charge transfer have the dominant role in the molecule-surface interaction, while the interaction between the transition metal and gold has a rather indirect influence over the physics of the molecule-surface system. A detailed analysis of the physical properties of the adsorbates at different geometric configurations allows us to propose qualitative models to account for all values of interface dipole charge transfer and magnetic moment of metal-phthalocyanines adsorbed on Au(111).

## Introduction

Metal phthalocyanines are highly symmetric organic molecules that incorporate a metal atom as the center of the organic ligand. Transition-metal (TM) phthalocyanines (TMPC) have been in the focus of scientific research for a long time^1^ because of application prospects in a wide number of fields, from photovoltaics^2^ to molecular electronics and spintronics^3–7^. The majority of these applications involve the adsorption of TMPC molecules on metal surfaces; in particular the adsorption of TMPC (TM = Cr to Cu) on Au(111) was widely investigated^8–15^ due to their chemical stability and potential applications.

The large adsorption energies (few eV) result from their size in combination with the flat adsorption geometry, which brings most atoms of the molecule in contact with the surface. The role of TM is revealed by the trend of binding energy for various TMPC’s; for example, for TMPC on Au(110) it was found that the binding energy follows the trend: CuPC < CoPC < FePC^16,17^.

The property of TMPC to form ordered structures depends on the balance between inter-molecular interactions arising at the molecule-surface interface. Attractive forces result mostly from van der Waals interactions; hydrogen bonds can also play a role, in the case of phthalocyanines with a partially halogenated periphery. Finally, the resulting dipole moments formed at surface are inducing parallel repulsive intermolecular interactions which compete with the attractive interactions in formation of self-assembled structures.

An important aspect influencing the TMPC assembly on surfaces include the match or mismatch between the molecular symmetry and the symmetry of the substrate. Depending on the system, such mismatch can lead to substrate-induced template effects, which affect the supramolecular arrangement as proven by STM investigations^18–24^. Such symmetry mismatch is also expected to play a role in the on-surface template synthesis of phthalocyanines, holding one of the top positions for future applications^25–30^.

In order to understand the role of this symmetry mismatch, DFT is a powerful tool due to its potential of providing information at nanoscopic scale: binding energy, geometric structure and electronic structure (e.g. HOMO-LUMO lineup with respect to Fermi level)^31–37^. The aim of the present investigation is to provide a model for the binding energy landscape formed by adsorbing TMPC on different positions on gold surface (TM = transition metals from Cr to Cu). The focus is put on monitoring the physical properties of adsorbed TMPC’s for different positions on top of a surface with hexagonal symmetry, i.e. Au(111).

In this purpose we have used exchange-correlation functionals adapted to van der Waals interactions combined with DFT + U corrections to the electronic structure of TM’s. Our data and models will provide useful tools to be used in the design and fabrication of self-assembled structures as well as for the synthesis of the covalently bonded layers of TMPC on noble metal surfaces.

## Methods

The simulations were performed using the Siesta code^38,39^ that uses norm-conserving pseudopotentials^40^ and expands the wave functions of valence electrons by linear combinations of atomic orbitals (LCAO). We used the van der Waals exchange-correlation functional of Berland and Hyldgaard (BH)^41^. During our tests we found that the BH functional correctly describes the geometric structure of the metallic bulks and surfaces which is a major advantage in the study of molecular adsorption^42^.

All systems were confined to a unit cells that allow the study of periodically Au(111) surface. We set the Au(111) surface to be parallel to XOY plane; the super-cell has a size of 7 × 7 Au atoms in XOY plane and include 3 atomic layers in the unit cell, with a total of 147 Au atoms. The length of the cell along the *OZ* axis was set to *L*_*Z*_ = 30 Å for all models in order to avoid the spurious influence of the electric charge from one cell to another. The bulk parameter for gold has the value of 4.08 Å (i.e. experimental value). We note that the theoretical value for bulk parameters produced by using BH functional and plane wave calculations was 4.1 Å^41^. Our tests on total energy versus lattice parameter lead to a value close to 4.095 Å. In other words, by using the experimental parameter for the bulk lattice we have an error that is below 0.5%.

In Supplementary material we present a ball-and-stick representation of the model used in calculations (i.e. gold slab and molecule). In order to test the validity of our model we have built a 3 × 3 supercell showing that the minimum distance between atoms in the periodic replica of the adsorbed molecule is around 8.5 Å. Consequently, we do not expect log-range interactions between periodic replica of the molecule to influence our results (see Supplementary material).

We used a 3 × 3 Monkhorst-Pack grid for the integrals in the Brillouin zone for the transversal direction while the periodicity along *Z* axis was modeled with a single k-point. We note that our tests indicates that the use of a 4 × 4 grid leads to a difference of 0.05 eV in the Kohn-Shamm energy (i.e. about 0.25 meV/atom). As basis sets, we used a double-zeta polarized basis set For Au atoms; a triple-zeta polarized basis set was used for molecule. The energy shift in LCAO basis set was 50 meV for all atoms. The value of 50 meV was chosen to be smaller than the standards SIESTA value (about 200 meV); this allow us to produce orbitals with a larger cutoff-radii in order to accurately simulate the long-range interactions. The grid used for the calculation of integrals and the representation of charge density and potentials was defined by its plane wave cutoff^38,39^; the value used in our calculations was 250 Ry.

The systems were relaxed by keeping the two bottom layers of gold pinned to their positions, allowing all other atoms to relax. The maximum gradient in the relaxed structure was 0.02 eV/Å. Additional details on the geometric structures and relaxation procedure for molecule are presented in the next section.

Despite their ability to describe metallic behavior, the exchange-correlation (xc) functionals used in DFT are plagued by one- and many-electron self-interaction error (SIE)^43^, a consequence of the mean-field models used in DFT. In particular the DFT treatment of 3*d* shells of transition metals, ask for corrections to compensate SIE.

One of the most widely employed method to correct SIE in classical approximate xc functionals is the so-called DFT + U approach^44^ (see the Supplementary material for details). We applied the DFT + U corrections to 3*d* orbital of TM in TMPC for all systems. We used *J* = 0.2 eV (i.e. corresponding to small hopping probability), while for *U* we employ the values computed from the linear response theory, indicated in ref.^45^ (see Supplementary material for complete list of values). For selected cases we also used the values of the best fit results presented in^45^. Throughout the paper we report the results for *U* extracted from linear response theory; we specifically indicate the cases where the results of the fit were used.

## Results and Discussion

### Geometric structures

**Figure 1:**
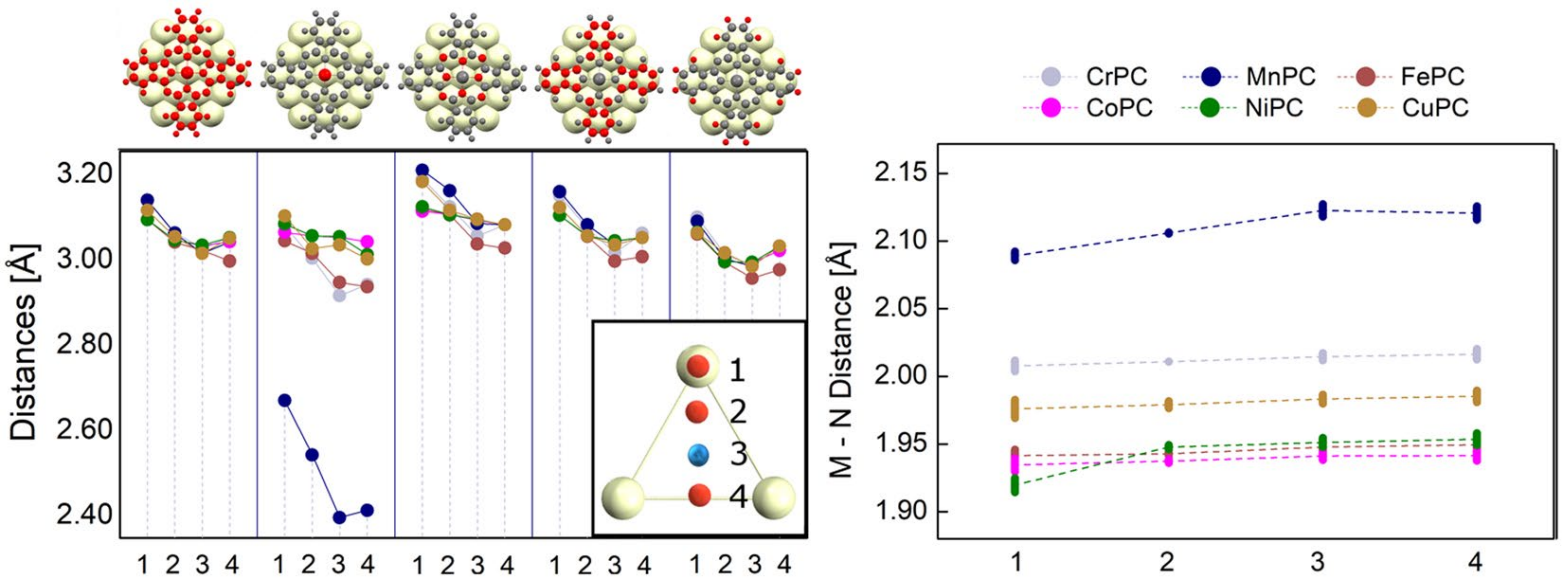
Left: graphic representation of the molecule-surface distance for all categories of atoms. Top-inset indicates from left to right: all molecule, transition metal, N, C and H. Bottom-inset: schematic representation of the positions of TM atom on top of Au(111) - labeled 1 to 4. Right: average value for nitrogen-metal distances parallel to the OX axis (i.e. formed by points 1–4). The differences between this value and the average distance parallel to OY axis are presented as error-bars.

Since Au(111) surface has a hexagonal symmetry, a relatively low number of points is sufficient to get information on the dependence of molecule-surface binding energy on the position of TM on top of Au(111). The four points indicated in the inset of Fig. 1 - left will provide information for a number of 10 points. The triangle is symmetric with respect to a C3 rotation axis perpendicular to the surface in its center (point 3 in Fig. 1 - left inset). For each TMPC we placed the metal atom on top of points 1 to 4 and we allow the metallic atom in TMPC to be relaxed only along OZ axis (i.e. perpendicular to the surface) while all other atoms in the molecule are free to fully relax their position.

Let us comment on the choice of restricting the degrees of freedom for TM to only OZ axis. The main cause for this is the complex shape of the potential energy surface for the PC-Au(111) system. Due to large size of the molecule, the system’s energy depends on a large number of degrees of freedom. Also, different types of symmetries are present in the system (hexagonal for the substrate, D4h for the molecule). All this leads to a big number of local minima for system’s energy, with values close to each other. Therefore, it is a good approximation to consider the structural relaxation of the PC’s adsorbed on Au(111) reduced to two independent relaxations: relaxation of the molecule’s internal structure (i.e. finding its Z-matrix) and relaxation of the molecule-surface distance. According to this model, a limitation for the degrees of freedom of TM will have practically no influence on the structural relaxation. In order to test this ansatz, we restart the structural relaxation of the systems from the structures reported in below, this time with no restrictions on the TM atom. This leads to energetic stabilization of few meV, which is a negligible energy for the molecule-surface system. Such result fully supports our simple model of two independent relaxations.

In Fig. 1-left we summarized the average values for distances between surface and specific categories of atoms in the molecule. They were computed by subtracted the average Z coordinate of gold atoms in the top layer of Au(111) from the average Z coordinate of the atoms indicated in the inset: TM, C, N and H atoms, respectively. We also present the average distance between molecule and gold surface. While there is no straightforward dependence between atom-surface distance and the strength of the interaction, the comparison between similar systems may be considered as a qualitative indicator for the strength of the molecule -surface interaction. Therefore, throughout the paper we consider that “interaction strength” is directly dependent on the distance between specific category of atoms and the surface. However, this should be seen in the context of this discussion.

For the average distance between the molecule and surface a decreasing trend between points 1 and 3 is present (see Fig. 1). We note that between points 3 and 4, there is no common trend for all systems: for FePC the molecule-surface distance decreases even more in position 4, while for the rest this effect is not present. All this suggests that the points 3 and 4 are energetically preferable for the molecule.

The largest difference between all systems occurs for MnPC: the Mn atom is located about 0.5 Å closer to the surface compared to the positions of all other metals. This indicates a qualitatively different bonding mechanism in MnPC, to be discussed in the next sections.

Moreover, the trends for metal-surface distances compared for points 3 and 4 show the presence of two distinct situations: the trend between points 1 and 3 is descendant for all systems. It remains descendant between 3 and 4 only for MnPC and CrPC. For all other molecules the distance molecule-surface is increasing when the TM is in point 4. This means that the metallic atom prefers the hollow position (point 3) for elements ranging from Fe to Cu; for Cr and Mn the geometric structure obtained at point 4 allows the maximization of interaction between TM and gold surface.

If we go to the panels summarizing the distances between categories of atoms (i.e. N, C and H) and surface we note a common behavior for all systems. For N we see a decreasing trend between the points 1 and 4, while for C and H the minimum average distance is obtained for point 3, which is the position preferred by the molecule. The full meaning of this is revealed in the section devoted to binding energy analysis.

The TMPC molecules have a D4h symmetry; consequently, the four metal-nitrogen distances are all equal. Upon adsorption we expect some distortion from this symmetric structure. The cause of the distortion originates in the molecule-surface interaction, on one hand, and the mismatch between hexagonal symmetry of the substrate on the other hand. This will lead to a slightly different interaction between nitrogen atoms and gold, depending on their relative positions. By following this assumption we can separate the four nitrogen atoms in two categories, according to nitrogen-gold distances and symmetry properties. Precisely, the two nitrogens placed on the axis formed by the four positions of the TM (axis 1–4) represent the first group while the other two, are placed on the axis perpendicular to that. Due to different gold-nitrogen distances we expect slightly different interactions with the surface for each of this category. Consequently, the D4h symmetry will be distorted. By estimating the distances between the nitrogens and central TM atom we can get an estimation of the influence of the surface upon molecule’s internal structure.

In Fig. 1-right we represent the distances parallel to OX while the differences between the distances parallel to OX and those parallel to OY are represented using error-bars style. We observe a common trend of increasing the metal-N distance on OX axis between points 1 and 4. This increase of the bond length may be associated to a weaker chemical bond. Nevertheless, this trend is negligible, with two exceptions: MnPC and NiPC. In these two cases the effect of weakening the chemical metal-N bond is likely to be present. Our second conclusion here is that the molecule’s deformation is minimal in the position corresponding to point 2; positions 1 and 3 typically lead to strongest deformation. This effect of mismatch between molecule and surface is maximized in the high symmetry points on top Au(111) (i.e. top and hollow configurations).

Due to the large differences between MnPC and other TMPC systems we perform a second set of calculations, using *U* = 4.0 eV (i.e. the result of fit to experiment for *U*^45^) in order to see the influence of *U* on geometric properties. Indeed, after the relaxation we found a planar structure for the adsorbed molecule, pointing out that the structural properties (in particular the gradients and forces) are extremely sensitive on *U* value. The average distances for each category of atoms for *U* = 6.1 eV and *U* = 4.0 eV, respectively are listed in Table 1. While Mn atom is still the closest to surface atom, the molecular deformation is significantly smaller. This has important consequences of the magnetic properties of the system.

**Table 1:** The molecule-surface distances, as defined in Fig. 1-left, for MnPC, all positions.

Pos.	*d*_*tot*_ [Å]	*d*_*Mn*_ [Å]	*d*_*N*_ [Å]	*d*_*C*_ [Å]	*d*_*H*_ [Å]
1	3.13	2.97	3.20	3.15	3.07
2	3.06	2.88	3.12	3.07	3.00
3	3.04	2.83	3.09	3.05	3.00
4	3.06	2.82	3.08	3.07	3.04
1	3.14	2.67	3.21	3.16	3.09
2	3.07	2.55	3.17	3.09	3.01
3	3.02	2.40	3.09	3.04	2.99
4	3.05	2.42	3.09	3.06	3.04

Top: values for *U* = 4.0 eV; bottom: values for *U* = 6.1 eV (represented in Fig. 1).

### Binding energy

The binding energy for the adsorbed molecules, Δ${\mathcal E}$, was computed by taking into account the BSSE effect as well as the molecular deformation upon adsorption^42,46^ (see Supplementary material for technical details).

**Figure 2:**
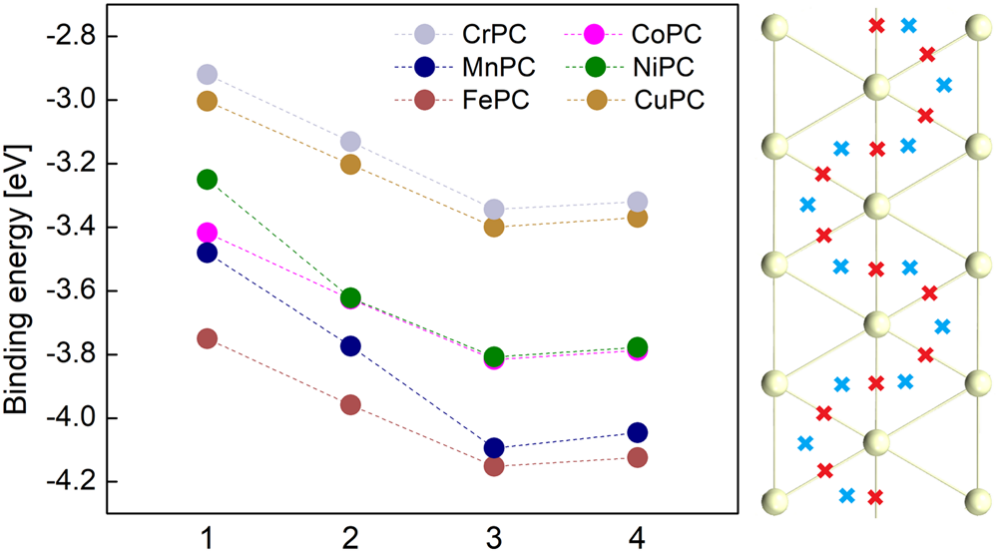
Left: Graphical representation of binding energy values Δ${\mathcal E}$, for all systems and relative molecule-surface positions. Right: sketch of the minimum energy path connecting possible equilibrium positions.

The summary of data for all systems is presented in Fig. 2. We found that the most stable structures are obtained for position 3 (metallic atom in the hollow position with respect to Au(111)). For CrPC and FePC we get the extreme values: 3.3 and 4.1 eV, respectively. We note a fluctuation of 25% of total binding energy can be obtained by interchanging the metals. Also, the trend of binding energies for TMPC on Au(110) is also valid here: CuPC < CoPC < FePC^16,17^.

From Fig. 2 we can see that the molecule-surface binding energies at the position with the lowest energy (i.e. 3 and 4) may be placed in three categories. The lowest binding energy was obtained for TM atoms with incomplete 4*s* shell (i.e. Cr and Cu). The gradual filling of the 3*d* orbital can also be seen in the trend of the binding energy: MnPC and FePC have very close and large binding energies. For CoPC and NiPC we get intermediate values.

A second conclusion arising form Fig. 2 is that the topological properties of the molecule-surface interaction energy are relatively similar for CrPC, CuPC, CoPC and FePC (differences between points 1 and 3 are all close to 0.4 eV). For MnPC and NiPC the energy difference between 1 and 3 reaches 0.6 eV. This corroborates with the hypothesis that the metal-N interaction is slightly weaker in the adsorbed MnPC and NiPC, as indicated in Fig. 1-right. Therefore we conclude that these modifications for Mn and Ni are probably caused by the weakening of metal-nitrogen bond upon the adsorption.

An important result is that for all systems the difference of binding energies between points 3 and 4 is around 0.05 eV. The minimum energy path connecting two equilibrium position for the molecules adsorbed on the surface is represented in Fig. 2 - right. For MnPC we note the presence of the deepest potential as well: yet the exact value is 0.05 eV. Our previous discussion on geometric parameters pointed out that Mn atom prefers position 3, which explains this extreme value. On the other hand, most of the metals prefer position 4 which ask for an analysis of the role of transition metal in the molecule-surface interaction.

By corroborating the results on binding energy with those obtained for distances it follows that the minimization of total energy for the metal in hollow position is most probably an effect of the carbon-surface and hydrogen-surface interaction. Indeed, from our distance analysis we note that the TM atom prefers point 4. The MnPC and CrPC are the only exceptions to this rule; for MnPC we note that this result in dependent on *U*. Indeed, for *U* = 4 the Mn also prefers point 4. Therefore we think that the TM’s contribution on the total binding energy should be an indirect one while no chemical bond is formed (i.e. direct interaction with the surface). The TM has an influence on the electronic structure of the molecular orbitals in PC; further interaction between PC and surface leads to different binding energies for each TMPC.

This assumption is further strengthened by the results on average distances presented in 1-left. They indicate that the difference between the average distances obtained by going from point 1 to 3 are smaller for TM’s than for C and H atoms, indicating the presence of a strong interaction between *π* rings and gold. For example for Cu, Co and Ni we get about 0.05 Å difference in average distance while for Fe and Cr the value is slightly larger than 0.1 Å. For C an H atoms, the differences are around 0.1–0.15 Å for all systems; their interaction to the surface is making a better distinction between points 1 and 3. Since this is van der Waals interaction, we conclude that this is the main factor responsible for the differences in binding energies between points 1 and 3. Moreover we see from the distance analysis that the hydrogens have the tendency to lay closer to the surface, indicating that they are interacting stronger to the surface. This effect is caused by the molecule-surface charge transfer, as we see in the next section.

The exception to this trends is the MnPC; in this case the metal-surface interaction is dominant, with almost 0.3 Å difference between points 1 and 3. This clearly shows the different behavior taking place in presence of a chemical interaction between TMPC and gold.

We note that the values reported in Fig. 2 are significantly larger compared to other values reported in literature (for example 0.88 eV for MnPc and MnFe, by Girovsky *et al*.^47^). This effect occurs in other comparative studies of molecule-Au(111) interaction: comparison between results produced by GGA and those obtained for functionals with van der Waals corrections reveal significant differences in binding energy. An example here can be found in the results of Mura *et al*.^48^. In their study, organic molecules with *π* rings are adsorbed parallel to the Au(111) surface. For medium size molecule such as naphthalene tetracarboxyldianhydride they get a binding energy of 0.1 eV using PBE xc functional while for vdw-DF corrected calculations the result is 1.31 eV^48^. In our view these large differences indicate the role of van der Waals interaction to the total binding energy.

Finally, let us note that the binding energies obtained for MnPC using *U* = 4 eV are about 1 to 3 percent larger as for *U* = 6.1. While the values are relatively small, it is interesting to note that for a larger distance Mn - surface (i.e. data for *U* = 6.1) we get a slightly larger binding energy.

### Electronic structure and magnetic properties

The electric dipoles at the surface can play an important role in the assembly mechanism of TMPC on gold. Therefore, we start our discussion on electronic structure and properties with the analysis of dipolar moments of TMPC adsorbed on Au(111). This late quantity was calculated as integral over the unit cell, ${\mathscr{D}}=\int \,\rho (\overrightarrow{r})\overrightarrow{r}dV$^38^ for all systems, after structural relaxation.

**Figure 3:**
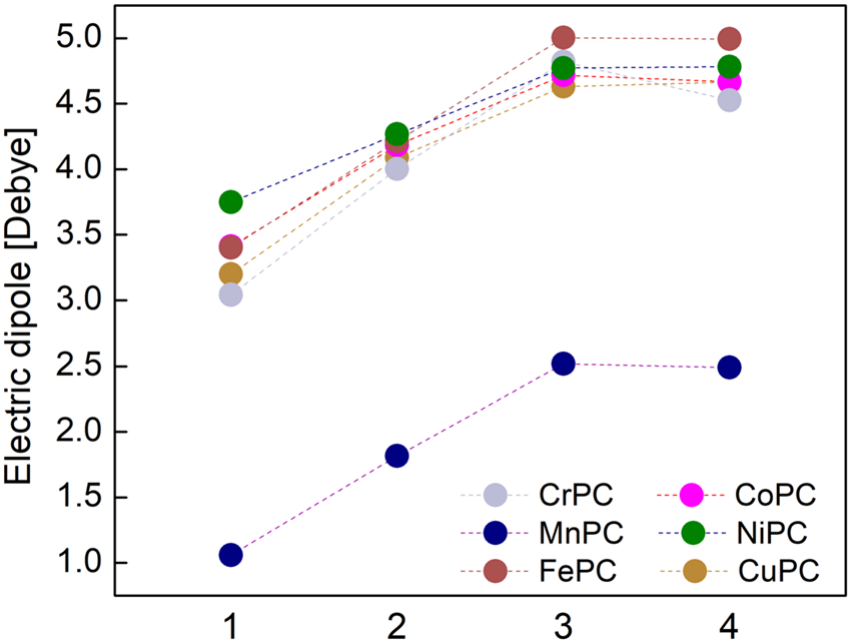
The dipolar moments for all TMPC’s and all molecule-surface relative positions.

Symmetry considerations indicates that all dipolar moments are oriented parallel to the OZ, (i.e. in the positive direction). Therefore, we report in Fig. 3 the absolute values of ${\mathscr{D}}$. The trend of dipolar moments between points 1 and 3 (i.e. top and hollow positions of TM) is similar for all systems. Smallest values occurs for top position of TM, then an increase with about 1.5 D occurs in hollow position. By going from point 3 to 4 we see only minimal changes in the dipoles of all systems. For NiPC we note the largest dipole at top position (around 3.8 D) and the smallest correction between points 1 and 3 (around 1D, compared to 1.5 D, the average value). The largest values occurs for FePC in positions 3 and 4. The absolute values of dipoles are similar for all systems, except for MnPC. In this case the values are with almost 2D smaller than for the other systems, indicating a different molecule-surface bonding mechanism. However, the results is strongly dependent on the *U* value. The calculations with *U* = 4 for MnPC produce a dramatically different value for ${\mathscr{D}}$, close to 6D (i.e. the largest values). This result is caused by two factors: the changes in the electronic structure and the relatively small shift of the Mn atom, compared to that obtained for *U* = 6.1.

In order to understand the influence of the dipolar moments in the self-energy process, we estimate the repulsion energy between adsorbed molecules, based on the results in Fig. 3. The minimum distance between metallic centers on the surface was set to 15 Å (i.e. the size of the molecule). The resulting dipolar interaction can be estimated at *kT*/10. This small value is the consequence of the dependence of dipolar interaction energy on 1/*r*^3^. While such value may play some role in the dynamics at room temperature, it is probably not one of the leading factors in the self-assembly mechanism.

Let us now search for the origin of the values presented in Fig. 3. They are determined by two factors: the molecule-surface distance and the molecule-surface charge transfer. We already saw that the trends of molecule-surface distances are opposite to that of the dipolar moments. It follows that the dependence of dipolar moments on position is determined by the charge transfer taking place between molecule and surface.

Charge transfer can have chemical origins or can be caused by the Pauli push-back of the electrons from the close shells and respectively from the surface^49,50^. A semiquantitative investigation of the charge migration can be done by using the quantity: ${\rm{\Delta }}\rho (\overrightarrow{r})={\rho }_{M+Au(111)}(\overrightarrow{r})-{\rho }_{M}(\overrightarrow{r})-{\rho }_{Au(111)}(\overrightarrow{r})$.

**Figure 4:**
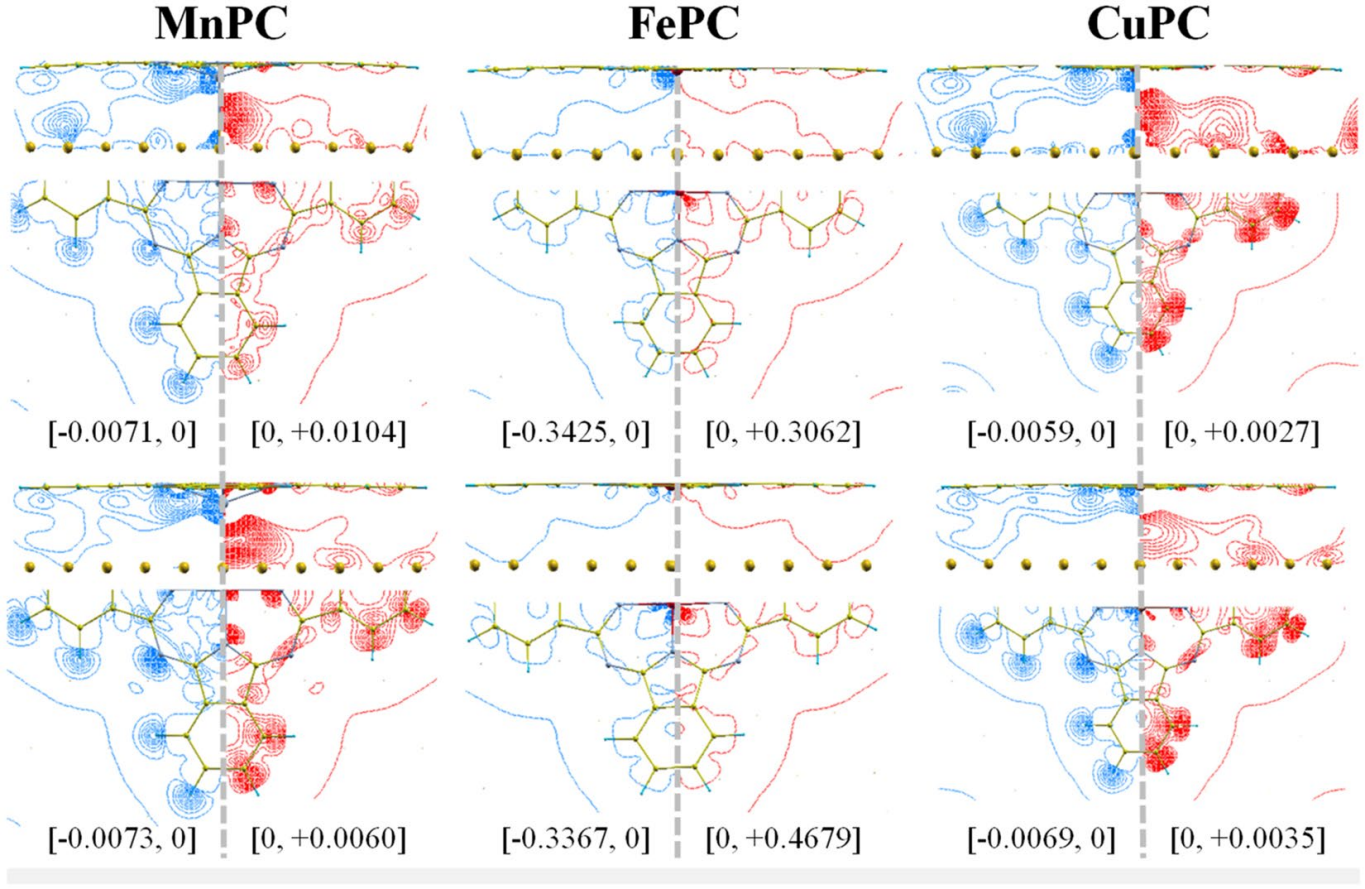
The contour plots of ${\rm{\Delta }}\rho (\overrightarrow{r})$ for selected models, represented using *N* = 30 lines of constant density. For each system we represent data in two planes containing the TM atom: first plane is perpendicular to the Au(111) surface (top of each figure) while the second includes the molecule (bottom of each figure). Positive contour lines are compared to negative contour lines for each system in the left/right half of each panel. Top panel gives results for TM on position 1 while the bottom panel presents the results for TM on position 3. Maximum/minimum values of the plot are in e/Bohr^3^ and their values are indicated on each plot.

The values of ${\rm{\Delta }}\rho (\overrightarrow{r})$ for three systems (M = Mn, Cu, Fe) are summarized in Fig. 4. They display two possible scenarios for charge redistribution upon adsorption: for CuPC and MnPC ${\rm{\Delta }}\rho (\overrightarrow{r})$ is distributed over the entire molecule while for FePC we found a very localized charge transfer around the iron atom. Let us investigate the two situations.

For Mn/CuPC the analysis of Fig. 3 shows that the hydrogen atoms transfer electrons toward the rest of molecule. The charge is pushed in two directions: in-plane, to carbons in benzene and out-of-plane, to the region placed below the metal in TMPC. The effect can be seen in the analysis of the average distances between carbons/hydrogens and surface (see Fig. 1): hydrogen atoms tend to be placed at 0.05 Å closer to the surface than carbons for all cases. Also, the effect can be seen by integrating the charge density around a hydrogen atom in the free molecule and comparing to the value in adsorbed state. This analysis shows a loss of 3 to 4 percent of the electronic population of hydrogen in adsorbed state compared to free molecule. Differences between positions 1 and 3 are significantly smaller (below 1 percent, see Supplementary material).

In order to understand the differences between MnPC and CuPC dipolar moments we note that the symmetry of the ${\rm{\Delta }}\rho (\overrightarrow{r})$ is similar for both molecules. From a quantitative perspective, larger values for ${\rm{\Delta }}\rho (\overrightarrow{r})$ occur in the case of MnPC. While negative values are roughly similar for both systems, the positive ones are about 2 times larger for MnPC. It follows that the large differences between the dipolar moments stems in the values of molecule-surface charge transfer, which in turn is influenced by the low position of Mn on top of Au(111), compared to that of Cu.

For FePC we see that the charge transfer takes place in a localized region (i.e. on the Fe atom) reaching values with almost two orders of magnitude larger as those obtained for MnPC. This is a consequence of changes in magnetic moment of adsorbed molecules compared to that of the free molecule, which is caused by the split of molecular orbitals in the crystalline field around TM atom. In other words, by inspecting the sum of magnetic moments in molecule and surface (as separate systems) we get different results from those on the molecule-surface system. The differences are caused by the magnetic properties of the molecule, since the surface is non-magnetic and are large for all systems, except MnPC and CuPC (see also Supplementary material).

Differences in magnetic moments cause large variations of electronic density (i.e. redistribution of electronic populations). This explains the large values of ${\rm{\Delta }}\rho (\overrightarrow{r})$ for FePC, for example. Therefore, in these cases it is difficult to draw further conclusions on the mechanism leading to the formation of surface dipole. In order to validate our discussion on charge migration from hydrogen to surface, we perform the analysis of ${\rm{\Delta }}\rho (\overrightarrow{r})$ for small values, comparable to those obtained for Mn and Cu. The results for FePC are presented in Supplementary material and they confirm the mechanism described for Mn and Cu.

The first step in our analysis of electronic structure was to investigate the values of the HOMO-LUMO gap (*γ*) and compare it to the results in literature^45,51^. Our data are summarized in Table 2.

**Table 2:** Value for HOMO-LUMO gap (*γ*) for TMPC as free molecules.

Molecule	CrPC	MnPC	FePC	CoPC	NiPC	CuPC
*γ*[*eV*]	1.53	0.32	1.44	1.47	1.5	1.45

Electronic structures of TMPC molecules were discussed by Liao and Scheiner^51^. Latter on the effect of correlation (i.e. DFT + U calculations) was investigated by Brumboiu *et al*.^45^. By corroborating the results of these two studies we note that in the absence of correlation, the HOMO-LUMO gap as well as the metal’s contribution to the frontier orbitals may vary. While in the work of Liao and Scheiner^51^ the maximum values of *γ* reach up to 1.96 eV (for CoPC), in the presence of DFT + U correction the results are in the range 1.3–1.5 eV^45^. The exception is MnPC with a value for *γ* around 0.4 eV. We note that our results in Table 2 are very close to these values.

We investigate the atomic contributions to HOMO-LUMO orbitals by integrating the projected density of states over each atomic state (i.e. contribution to LCAO wavefunction) at energies corresponding to HOMO-LUMO eigenvalues of the Hamiltonian. We found that the HOMO of free molecules consist mainly in 2*pz* orbitals of carbons 2- and 5- in pyridine rings (the carbons in the nitrogen vicinity), while for LUMO the contributions of 2*pz* orbitals in nitrogen atoms are also important. Atomic orbitals of TM are present in HOMO for MnPC and FePC; this is similar to the results presented in^45^, while in^51^ the metallic atom was found to contribute to HOMO. The differences are the consequence of the DFT + U correction; its effect is to shift the occupied *d* orbitals of the transition metal toward lower energies, therefore removing their contribution from HOMO Table 3.

**Table 3:** Magnetic moments for TMPC free and adsorbed in positions 1/3.

Model	CrPC	MnPC	FePC	CoPC	NiPC	CuPC
*μ*_1_	3.88/3.89	4.88/4.87	1.92/1.95	0.95/0.91	0.00/0.00	1.00/1.00
*μ*_2_	4.00/0.01	5.00/5.00	3.99/3.99	1.00/0.00	0.0/2.00	1.00/1.00

Top: adsorbed molecules, *μ*_1_; bottom: single molecule at the adsorption geometry, *μ*_2_.

The contributions of TM are different in the two cases. In FePC we found that HOMO is essentially formed by 3*dxz* and 3*dyz* orbitals of iron. LUMO was found to be similar for all molecules, as discussed above (see also Liao and Scheiner^51^). On the other hand, the MnPC has an important contribution of 3*dxz* orbital to LUMO (about 12%) - see Supplementary material for detailed graphs of PDOS. This leads to different behavior of the two molecules adsorbed on gold surfaces.

**Figure 5:**
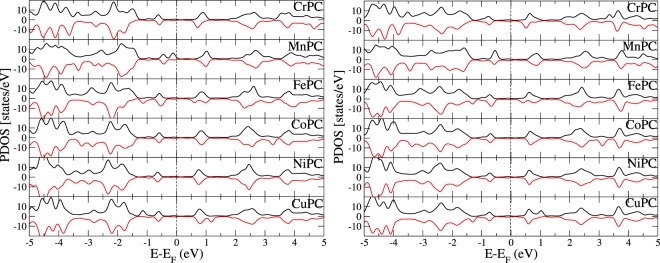
The density of states projected over the adsorbed molecule; left: position 1, right: position 3. Metal’s Fermi level is set to zero.

The densities of states of the adsorbed molecule for all positions of TM are summarized in Fig. 5. The HOMO-LUMO gaps in adsorbed state are close to those obtained in the isolated molecules (only few percent smaller due to the broadening of the DOS because of the interactions with Au(111) substrate). The HOMO of adsorbed molecules lies between −0.5 and −1.0 eV with respect to Au(111) Fermi level, while LUMO is found at 0.5 eV.

Experimental data for the frontier orbitals for TMPC or similar systems indicate values that are close to our results, as follows: the electronic states for CuPC adsorbed on Au(111) were found at −0.4 eV and −1.46 eV^9^. For Ni porphyrin complex on Au(111) a molecular state was reported at −1.2 eV^52^ while for CoPC, the value of −0.7 eV was reported^16,17^. For FePC on Au(111) the value was −1.0 eV^53,54^ while for MnPc on Au(110) molecular states were found at −0.3 and −0.9 eV^55^.

As a general rule, the atomic contribution to HOMO-LUMO of adsorbed molecules are unchanged upon adsorption; frontier orbitals are placed right below (HOMO) and above (LUMO) with respect to Fermi level of Au(111). The exception is MnPC, where the HOMO-LUMO in the adsorbed molecule shows the presence of different atomic orbitals in the adsorbed molecule, compared to the isolated MnPC. The cause is the position of LUMO, just below metal’s Fermi level; consequently, HOMO and LUMO are strongly perturbed (see also Supplementary material for more details on PDOS of MnPC). This explains the systematic differences we found in the properties of MnPC with respect to other molecules. The occupation of LUMO is responsible for the weakening of the metal-nitrogen bond as well as for the strong deformation and longer Mn-N bonds reported above. On the other hand, chemical interaction accounts for the strong binding energy.

For FePC we have found small variations in the LCAO coefficients of the atomic orbitals in adsorbed state, compared to those for free molecule. Nevertheless the effect is far weaker than for MnPC; HOMO-LUMO of adsorbed FePC are positioned symmetric with respect to metal’s Fermi level. The effect is not responsible for the large binding energy of FePC. Instead, we found that HOMO of iron is closer in energy to Au(111) Fermi level, and has contributions from 3d orbital which is a possible explanation for the strong bonding energy. All other molecules have density of states with peaks assigned to TM at deep energies, which makes the interaction to gold indirect, as discussed above (i.e. via the molecular orbitals and charge transfer effects).

Our values for magnetic moments are relatively close to those reported in^45^ for free molecules; the largest difference occurs for MnPC (3.5 versus 4.88 *μ*_*b*_). However the value of 4.88 *μ*_*B*_ for MnPC is not consistent with experimental value of 3.8 *μ*_*b*_ reported by Girovsky *et al*.^47^. To explain this result we observe that 4.88 *μ*_*b*_ is close to what we can expect for a single Mn atom (i.e. Hund rule for 3*d*^5^ electronic configuration). We think that the strong distortion after the relaxation of MnPC leads to a geometric structure where all effects of crystal-field splitting in MnPC are removed. Consequently, the electronic structure of Mn atom in adsorbed MnPC is close to that of a single Mn atom, i.e. five degenerate 3*d* spins.

In order to further clarify this point, we performed a single point calculation (i.e. no relaxation) of a flat MnPc molecule on top of Au(111), at heights 3.2, 3.4 and 3.6 Å. The resulting magnetic moments are all close to 3.7 *μ*_*B*_, supporting our ansatz that in the first series of results the crystal-field splitting is removed by the wrong geometric structure.

Moreover, our second set of calculations for MnPC (i.e. using *U* = 4.0 eV) lead to a geometric model consisting on an almost flat MnPC molecule adsorbed on top of Au(111). For positions 1 to 4 we found magnetic moments around 3.7 *μ*_*B*_, which is close to experimental results in^47^. The complete set of data is reported in the Supplementary material.

To conclude, we observe that for most of TM’s the data obtained from linear response provide results in agreement with existing literature; the exception is MnPC, where the value of *U* = 4.0 eV (i.e. fit to experiment) is the correct one to be used for DFT + U calculations. Most probably, the cause of failure for *U* = 6.1 are the wrong gradients and forces computed by using the DFT + U.

While the molecular internal degrees of freedom are different for positions 1 to 4 (see Fig. 1) we get similar values for magnetic moment, *μ*. Calculations on the isolated molecules at adsorption geometries shows relatively important variations of magnetic moment with respect to those obtained for the adsorbed molecule: they are between 0.1 to 1 *μ*_*B*_ and occur for all systems, except the CuPC. This indicates that the crystal-filed splitting of 3*d* orbitals alone is not enough to explain the magnetic moments of the adsorbed molecules. The hypothesis of interaction between molecule and metallic surface should be taken into account in order to explain the relatively constant magnetic moment of the adsorbed molecule in all four positions.

## Conclusion

In summary, we have presented first-principles results for adsorption of metal-phthalocyanine (metal = Cr to Cu) on Au(111) by using DFT. Our analysis was performed using van der Waals adapted exchange-correlation functionals, while the strong correlations in *d* orbitals of the transition metals were taken into account by the DFT + U method.

Four positions of the metallic atoms on top of Au(111) were investigated for each molecule in order to understand the translation of the molecules on the surface. Based on values of binding energy and molecule-surface geometry we conclude that van der Waals interaction has the decisive contribution to adsorption while the metal-surface interaction is rather weak. This conclusion is supported by the values of the binding energies as well as by the properties of the frontier orbitals in free and adsorbed molecules. Nevertheless, the molecule-surface binding energies can be correlated to the filling of 3*d* orbital in transition metal: MnPC and FePC display strongest interactions while smaller interaction occurs for CuPC.

The dependence of calculated properties on the position of transition metal on top of Au(111) is similar for all systems. For the binding energy we found a smooth dependence, with minima at hollow position and saddle points at bridge position; the energy barrier between two positions is typically less than 0.05 eV. For the dipolar moments we have found large differences (i.e. around 1.5 D) between the top and hollow positions while the maximum values were around 4 D. At the origin of these values is the the molecule-surface charge transfer: the electrons are pushed along a path starting from hydrogen atoms continuing to carbons to by finally transferred to the surface. We have shown that the crystal field splitting is not sufficient to explain magnetic properties of the adsorbed molecules; the magnetic polarization in the gold surface should be taken into account to explain the data.

Our study opens up new prospects for potential applications in the controlled manipulation of the metal-phthalocyanine on surface and also for the methodologies currently used in theoretical investigations of the molecules adsorbed on metallic surfaces.

## Electronic supplementary material

Supplementary material
