# Supplementary material for: Translation of metal-phthalocyanines adsorbed on Au(111): from van der Waals interaction to strong electronic correlation
Source: Sci Rep. 2018 Aug 24;8:12728. doi: 10.1038/s41598-018-31147-5 (PMC6109120; doi:10.1038/s41598-018-31147-5)
Supplement: Supplementary file 1 — Supplementary material [file 41598_2018_31147_MOESM1_ESM.pdf]

SUPPLEMENTARY INFORMATION

Translation of metal-phthalocyanines adsorbed on Au(111): from van der Waals interaction to strong electronic correlation

C. Morari and L. Buimaga-Iarinca*

National Institute for Research and Development of Isotopic and Molecular Technologies (NIRDIMT), 65-103 Donath, Ro-400293, Cluj-Napoca, Romania

E-mail: cristian.morari@itim-cj.ro

Computational details

The binding energy for an adsorbed molecule is

$$\Delta\mathcal{E} = E_{tot} - E_{mol} - E_{surf} \tag{1}$$

where E_{tot} is the total energy of the relaxed molecule-surface system, E_{mol} and E_{surf} are the total energies of the relaxed free molecule and surface, respectively. The main criticism against this formula is related to the well-known BSSE effect[1,2] that produces an overestimation of the binding energy due to the orbital overlap in the region between the molecule and surface. In order to get rid of this effect we compute the interaction en-

ergy in two steps. First, we calculate the BSSE corrected interaction energy between the molecule and surface, expressed as

$$\Delta E_1 = E_{MS}^{MS} - E_{M}^{MS} - E_{S}^{MS} \tag{2}$$

where M denotes the molecule, S the surface. Superscript index represent the geometric configuration (i.e. relaxed molecule-surface system) while the subscript index represent the atoms replaced by corresponding 'ghost atoms' (see reference[2] for further details.) We note that Eq. 2 represent the binding energy if the geometry of the adsorbed molecule suffer little deformation in the adsorption. In general, the geometric structure of adsorbed molecule has a different geometry compared to the geometric structure of the molecule in vacuum. This will add a positive component to the total energy of the molecule-surface system, with the value

$$\Delta E_2 = E_A - E_V \tag{3}$$

where E_V and E_A are the total energies of the molecule in vacuum (i.e. relaxed structure) and adsorbed (i.e. the geometric structure obtained in the adsorbed state). A similar expression occurs for the atoms in the surface. By combining the energies from the Equations 2 and 3, we get the best estimation for the binding energy computed in the LCAO framework.

The electronic correlation in the localized d orbitals of central atom of MPC leads to an energetic correction that is calculated by using DFT+U corrections as implemented in SIESTA.[5] DFT+U Hamiltonian has the form:

$$\hat{H} = \frac{\bar{U}}{2} \sum_{m,m',\sigma} \hat{n}_{m,\sigma}\hat{n}_{m',-\sigma} + \frac{\bar{U} - \bar{J}}{2} \sum_{m \neq m',\sigma} \hat{n}_{m,\sigma}\hat{n}_{m',\sigma} \tag{4}$$

where the summation runs over the projections of the orbital momentum (m and m') while $\bar{U}$ and $\bar{J}$ are the spherically averaged matrix elements for the screened on site Coulomb

and hopping interactions respectively. In terms of Kohn-Shamm eigenvalues ϵ_j, this produces the total energy:

$$E_{DFT+U} = E_{DFT}[\{\epsilon_j\}] + \frac{\bar{U} - \bar{J}}{2} \sum_{l,j,\sigma} \rho^{\sigma}_{l,j} \rho^{\sigma}_{jl} \tag{5}$$

where $\rho^{\sigma}_{l,j}$ is the density matrix of the manifold taken into account.

The DFT+U projectors used to calculate the local populations for the a Hubbard-like term are the exact solutions of the pseudoatomic problem which are cut using a Fermi function.[3,4,6]

Table S1: The values of U used in calculations. For all systems J was set to 0.2 eV.

System	CrPC	MnPC	FePC	CoPC	NiPC	CuPC
$U[eV]$	4.0	6.1	4.4	6.1	8.2	3.7

Geometric properties

The quality of the slab model for gold was tested by performing three tests for clean gold surface. First, we use the same approach as in the paper (i.e. three layers of gold, top layer is relaxed while the bottom layers are pinned to bulk position) - model M3-1. Next, we use the same geometric model, but we allow all atoms to relax - model M3-2. Finally, we take a ten layer model for the slab, and allow all atoms to relax - model M10. The lateral size of all these model is dictated by the bulk parameter (4.08 Å) and the periodic boundary conditions on Au(111) surface. The size on Z axis (i.e. perpendicular to surface) was 30 Å for the first two models and 60 Å for the last one, allowing a separation of over 25 Å between periodic replica of the slab. The distances between layers 1 and 2 (from top to bottom) as well as the distance between layers 2 and 3 are summarized in the table bellow. It can be seen that the model used in our calculations (i.e. M3-1) produces results the are almost identical to model M10.

Table S2: The distances between top layers of atoms in the gold slab, for three models for the gold slab. The models are explained in the text; d_{1-2} and d_{2-3} are expressed in Å .

Distance	M3-1	M3-2	M10
d_{1-2}	2.456	2.479	2.422
d_{2-3}	2.356	2.464	2.359

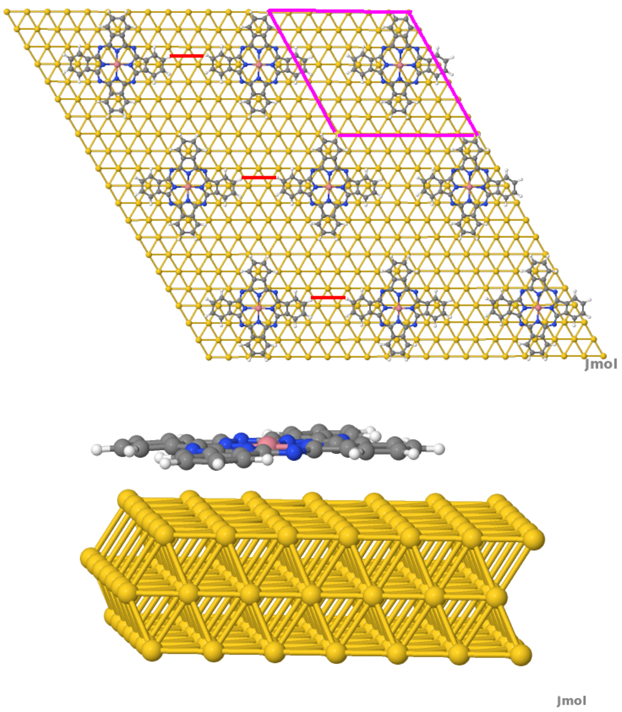

Figure S1: Top: Schematic representation of a 3×3 supercell for the adsorption model used in our calculation. We use magenta to indicate the atoms in the unit cell. Red lines indicate the minimum distance between atoms in the supercell. The length of these lines is ≈ 8.5 Å ensuring the negligible interaction between periodic replicas of the adsorbed molecule. We represent only the top layer of gold atoms for simplicity. Bottom: 3D graphic representation of the molecule - slab model used in calculation (the area indicated by magenta borders on top part of the picture).

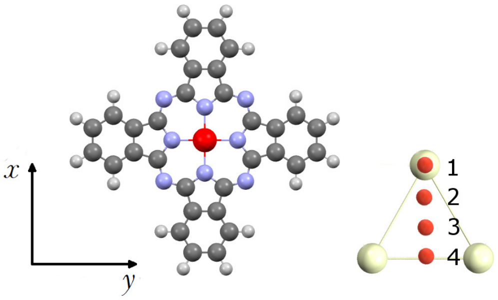

Figure S2: Schematic representation of MPC on top of Au(111) - left and the labeled positions of TM atom in top of Au(111). The Cartesian coordinate system is also represented for clarity.

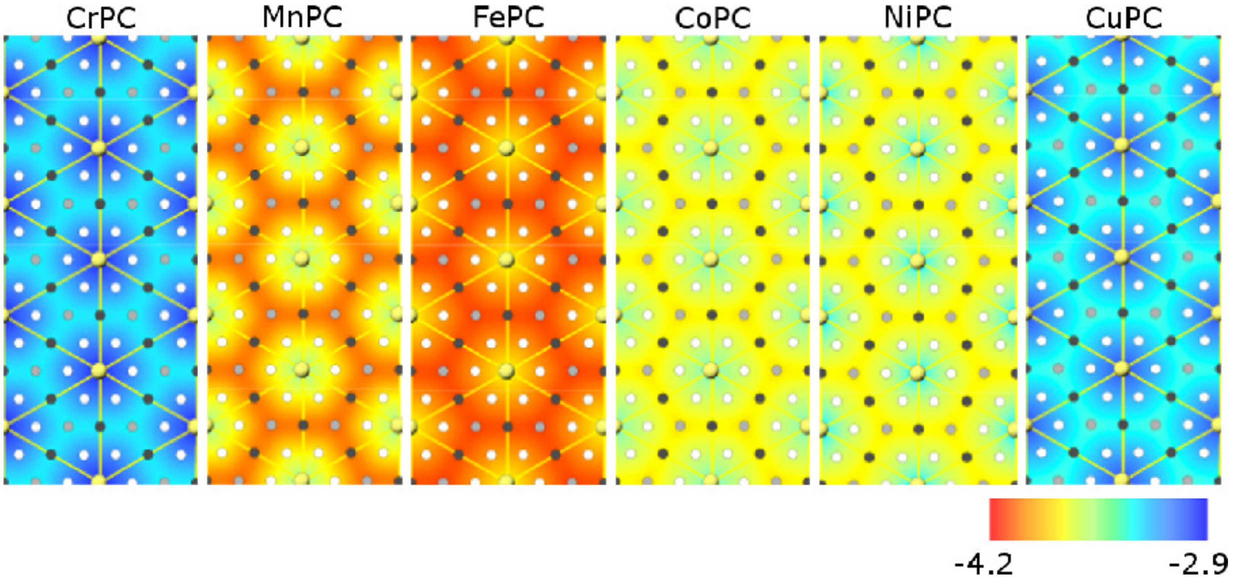

Figure S3: Energy map produced by fitting the results of the molecule-surface binding energies analysed in the text, for all M-PC systems. The color code is indicated in the bottom-right corner; values are in eV.

Table S3: Distances Metal-Nitrogen: D_x - average of the two distances parallel to the OX axis; D_y - average of the two distances parallel to the OY axis (see Figure 2. From top to bottom, positions 1 to 4. Last line: average values for the molecule in vacuum. In this last case the typical value for differences is around 1e^{-3} Å . The only exception is MnPC; in this case we see a difference of about 2e^{-2} Å between D_x and D_y for the molecule in vacuum.

	CrPC	MnPC	FePC	CoPC	NiPC	CuPC
D_x	2.008	2.089	1.941	1.935	1.919	1.976
D_y	2.012	2.092	1.946	1.939	1.925	1.983
D_x	2.011	2.106	1.943	1.937	1.948	1.979
D_y	2.011	2.105	1.944	1.939	1.949	1.982
D_x	2.014	2.122	1.947	1.941	1.951	1.983
D_y	2.012	2.118	1.9447	1.938	1.948	1.980
D_x	2.016	2.121	1.949	1.941	1.953	1.985
D_y	2.012	2.116	1.947	1.938	1.949	1.981
D_0	2.005	1.953	1.938	1.935	1.919	2.004

Table S4: Average distances along OZ axis between the atoms in Au(111) surface and different categories of atoms in molecule: all atoms (mol), metal (M), N, C and H. In each row are given the results for positions 1 to 4. For example Cr-1 = Cr is on top of point 1 in Figure 1. The second column indicated the maximum distance between Z coordinates of the atoms in molecule, ΔD. All values are in Å.

Model	D_{mol}	ΔD	D_M	D_N	D_C	D_H
Cr-1	3.14	0.10	3.08	3.19	3.15	3.10
Cr-2	3.07	0.14	3.01	3.18	3.08	3.02
Cr-3	3.03	0.10	2.92	3.06	3.02	2.99
Cr-4	3.06	0.10	2.95	3.09	3.07	3.04
Mn-1	3.14	0.47	2.67	3.21	3.16	3.09
Mn-2	3.07	0.51	2.55	3.17	3.09	3.01
Mn-3	3.02	0.61	2.40	3.09	3.04	2.99
Mn-4	3.05	0.63	2.42	3.09	3.06	3.04
Fe-1	3.10	0.11	3.05	3.12	3.11	3.06
Fe-2	3.04	0.17	3.02	3.11	3.06	3.00
Fe-3	3.03	0.10	2.95	3.04	3.00	2.96
Fe-4	3.00	0.09	2.94	3.03	3.01	2.98
Co-1	3.10	0.10	3.07	3.12	3.11	3.07
Co-2	3.05	0.17	3.06	3.11	3.06	3.00
Co-3	3.04	0.09	3.06	3.10	3.05	3.00
Co-4	3.05	0.09	3.05	3.09	3.06	3.03
Ni-1	3.10	0.10	3.09	3.13	3.11	3.07
Ni-2	3.05	0.17	3.06	3.11	3.06	3.00
Ni-3	3.04	0.10	3.06	3.10	3.05	3.00
Ni-4	3.06	0.08	3.02	3.09	3.06	3.04
Cu-1	3.12	0.13	3.11	3.19	3.13	3.07
Cu-2	3.06	0.12	3.03	3.12	3.06	3.02
Cu-3	3.02	0.10	3.04	3.10	3.04	3.00
Cu-4	3.05	0.08	3.01	3.09	3.06	3.04

Electronic structure and charge transfer

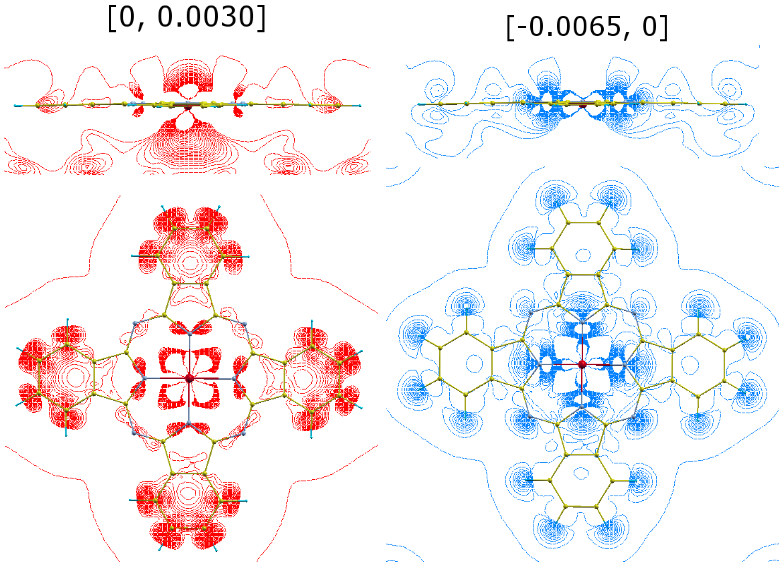

Figure S4: Contour plots for $\Delta\rho$ at small values, for Fe-PC.

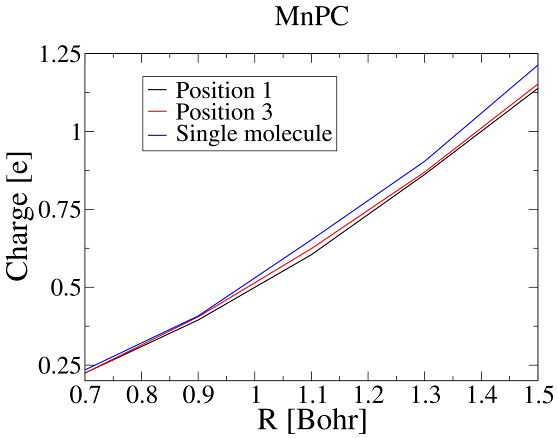

Figure S 5: Results for the integral $\int_0^{R0} \rho(\vec{r}) d\vec{r}$ for a hydrogen atom in MnPc as free molecule and adsorbed in positions 1 and 3. For $R0$ we took values between 0.7 and 1.5 Bohr.

Table S5: Top: maximum/minimum values for $\Delta\rho$ for 1/3 positions for all systems. A factor of 10^{-2} e/Bohr^2 has to be multiplied to all values. Bottom: magnetic moments of the molecules at adsorption geometry for 1/3 orientations expressed in Bohr.

Model	Cr-Pc	Mn-Pc	Fe-Pc	Co-Pc	Ni-Pc	Cu-Pc
V_{max}	0.43/48	1.01/0.83	53/54	44/0.18	0.33/39	0.31/0.36
V_{min}	-1.28/-68	-1.85/-1.19	-42/-41	-57/-48	-1.94/-44	-0.71/-0.83
spin	4.00/0.01	5.00/5.00	3.99/3.99	1.00/0.00	0.0/2.00	1.00/1.00

Table S6: The values of magnetic moments and dipoles for MnPc on top of Au(111) (at adsorption geometry) for three distances between MnPC and Au(111) (d), for $U = 6.1$ eV.

d [Å]	3.2	3.4	3.6
μ [μ_B]	3.71	3.81	3.86
$\mathcal{D}$[D]	7.27	7.51	7.86

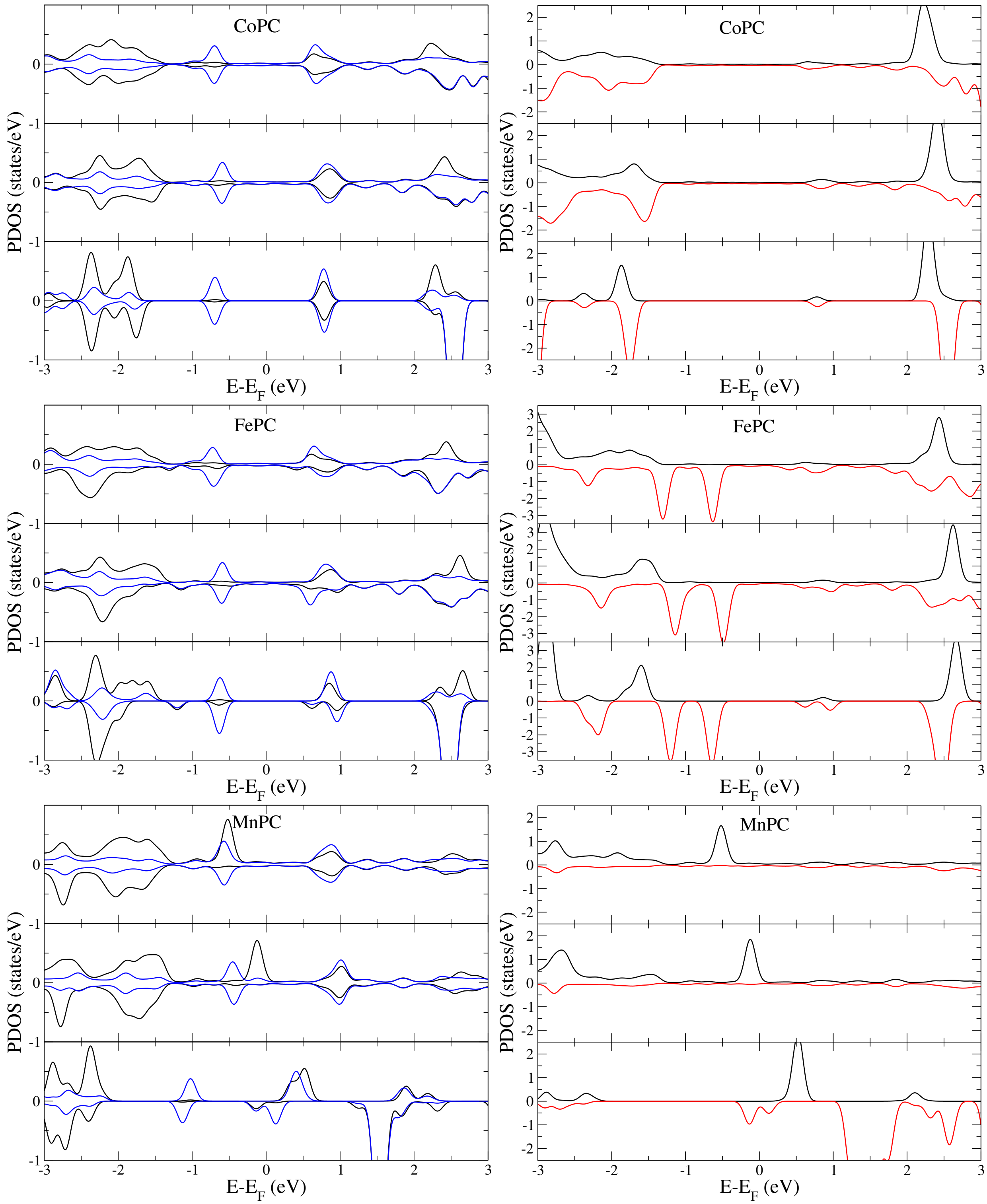

Figure S6: Left: Density of states projected over the 2,5-C atom in pyridine (blue line) and 1-N, respectively (black line). Right: Density of states projected over the central metallic atom MPC, for M=Fe, Co, Mn. Spin-up and down are given according to their orientation. From bottom to top, the panels are: free molecule, position 1 and 3. Fermi level is set to zero.[7]

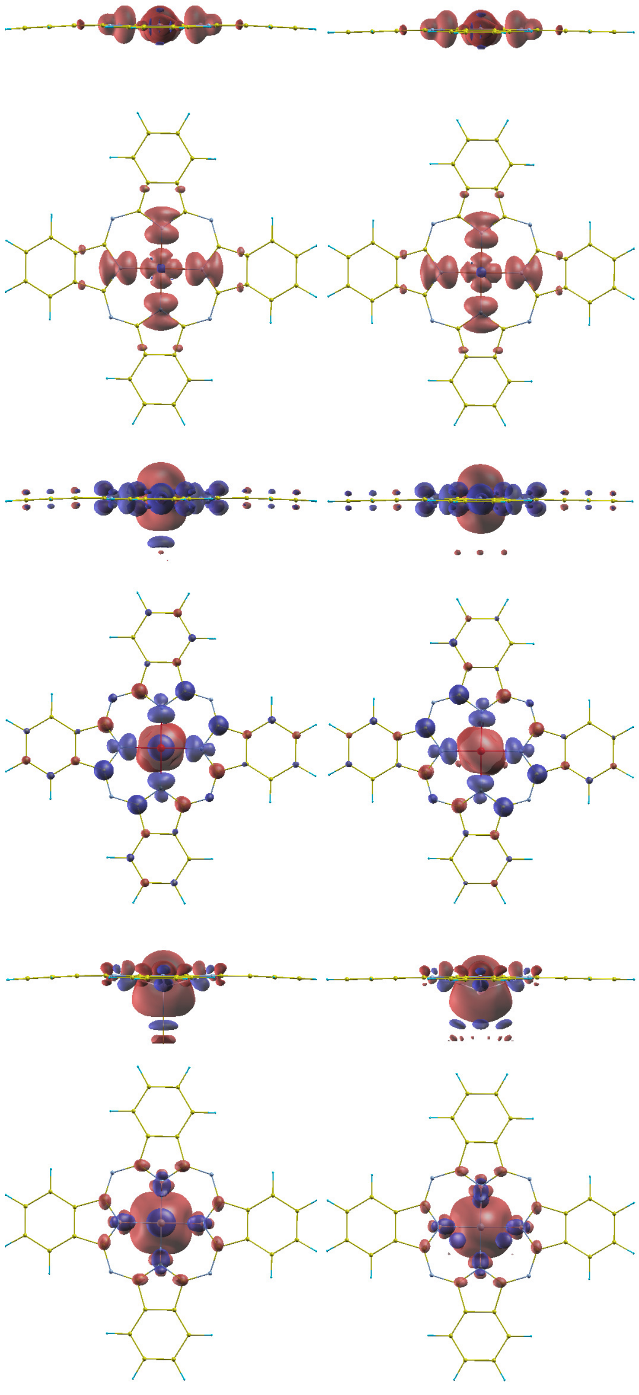

Figure S7: Spin density, $\sigma(\vec{r}) = \rho_{up}(\vec{r}) - \rho_{dn}(\vec{r})$ expressed in e/Bohr^3 , for CuPC (first two panels), FePC (second two panels) and MnPC (bottom panels). Left/right - position 1/3/ of the TM on top of Au(111). blue -negative; red - positive.

Calculations with $U = 4$ eV for MnPC

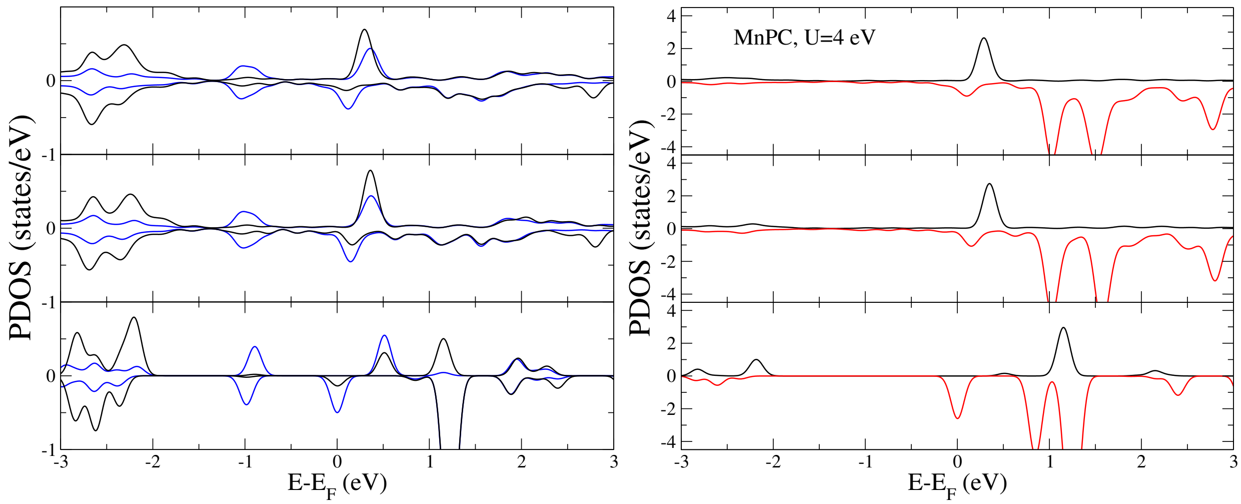

Figure S8: Density of states for MnPC, $U = 4$ eV, for spin up/down. Left: C (blue line) and N (black line), respectively. Right: Mn atom. Bottom to top, in each graph: free molecule, position 1 and 3.

Table S7: Dipolar, magnetic moments and binding energy ($\mathcal{D}$, μ , $\Delta\mathcal{E}$) for MnPC adsorbed in positions 1 to 4. Left: $U = 6.1$ eV; right: $U = 4$ eV.

Position	1	2	3	4	1	2	3	4
$\mathcal{D}$ [D]	1.06	1.83	2.52	2.49	6.62	6.74	6.80	6.89
μ [μ_B]	4.87	4.88	4.88	4.89	3.86	3.72	3.65	3.67
$\Delta\mathcal{E}$ [eV]	3.48	3.77	4.09	4.06	3.85	4.01	4.16	4.13

References

(1) Buimaga-Iarinca, L.; Morari, C. Adsorption of Small Aromatic Molecules on Gold: a DFT Localized Basis Set Study Including van der Waals Effects. *Theor. Chem. Acc.* **2014**, 133, 1502.

(2) Boys, S.F.; Bernardi, F. The calculation of Small Molecular Interactions by the Differences of Separate Total Energies. Some Procedures With Reduced Errors. *Mol. Phys.* **1970**, 19, 553 - 566.

(3) Ordejón, P.; Artacho, E.; Soler, J. M. Self-Consistent Order-N Density-Functional Calculations for Very Large Systems. *Phys. Rev. B* **1996**, 53, R10441 - R10444.

(4) Soler, J. M.; Artacho, E.; Gale, J. D.; García, A.; Junquera, J.; Ordejón, P.; Sánchez-Portal, D. The SIESTA Method for Ab Initio Order-N Materials Simulation. *J. Phys.: Condens. Matter.* **2002**, 14, 2745 - 2779.

(5) Dudarev, S. L.; Botton, G. A.; Savrasov, S. Y.; Humphreys, C. J.; Sutton, A. P. Electron-Energy-Loss Spectra and the Structural Stability of Nickel Oxide: An LSDA+U Study. *Phys. Rev. B* **1998**, 57, 1505 - 1509.

(6) See also the SIESTA distribution site, https://launchpad.net/siesta

(7) For molecule we use the ”Fermi level” provided by Siesta, which has no immediate physical meaning. Nevertheless, it is placed between the HOMO and LUMO orbitals, allowing a clear distinction between the frontier orbitals
